# School Health: an essential strategy in promoting community resilience and preparedness for natural disasters

**DOI:** 10.3402/gha.v8.29106

**Published:** 2015-12-17

**Authors:** Kenzo Takahashi, Mitsuya Kodama, Ernesto R. Gregorio, Sachi Tomokawa, Takashi Asakura, Jitra Waikagul, Jun Kobayashi

**Affiliations:** 1 Japanese Consortium for Global School Health Research, Okinawa, Japan; 2 Teikyo University Graduate School of Public Health, Tokyo, Japan; 3 Global Cooperation Institute for Sustainable Cities, Yokohama City University, Kanazawa, Japan; 4 Department of Health Promotion and Education, College of Public Health, University of the Philippines Manila, Manila, the Philippines; 5 Faculty of Education, Shinshu University, Nagano, Japan; 6 Department of Education, Tokyo Gakugei University, Tokyo, Japan; 7 School Health Promotion Unit, Faculty of Tropical Medicine, Mahidol University, Bangkok, Thailand; 8 Department of Global Health, School of Health Sciences, University of the Ryukyus, Okinawa, Japan

**Keywords:** disaster risk reduction, school health, preparedness

## Abstract

**Background:**

The Third UN World Conference on Disaster Risk Reduction recommended the implementation of the Sendai Framework for Disaster Risk Reduction 2015–2030, which aims to achieve substantial risk reduction and to avoid various disaster-associated losses, including human lives and livelihoods, based on the lessons from the implementation of the Hyogo framework. However, the recommendations did not lay enough stress on the school and the Safe School Concept, which are the core components of a disaster response.

**Objective:**

To raise the issue of the importance of schools in disaster response.

**Results:**

For human capacity building to avoid the damage caused by natural disasters, we should focus on the function of schools in the community and on school health framework. Schools perform a range of functions, which include being a landmark place for evacuation, acting as a participatory education hub among communities (students are usually from the surrounding communities), and being a sustainable source of current disaster-related information. In 2007, the Bangkok Action Agenda (BAA) on school education and disaster risk reduction (DRR) recommended the integration of DRR into education policy development, the enhancement of participatory mechanisms to improve DRR education, and the extension of DRR education from schools to communities. Based on our discussion and the recommendations of the BAA, we suggest that our existing challenges are to construct a repository of disaster-related lessons, develop training materials based on current information drawn from previous disasters, and disseminate the training to schools and communities.

**Conclusions:**

Schools linked with school health can provide good opportunities for DRR with a focus on development of school health policy and a community-oriented participatory approach.

At present, the world faces a large number of natural and man-made disasters, which have massive negative effects on the people in the affected countries, especially those in Asian countries (1). The Third UN World Conference on Disaster Risk Reduction was held in Sendai, Japan, from March 14 to 18, 2015. Approximately 6,500 participants from 187 member states of the United Nations were in attendance. In the conference, several recommendations were made, including that the Sendai Framework for Disaster Risk Reduction 2015–2030 (Sendai Framework) be implemented (2, 3). We recognize the Sendai Framework as a comprehensive evolutionary form of the Hyogo Framework for Action (HFA) of 2005–2015 (4). A core component of the HFA, which was recognized as a highly important for DRR, was the ‘use of knowledge, innovation, and education to build a culture of safety and resilience at all levels’. After the HFA had been in place for several years, a midterm review in 2011 identified the existence of ‘a significant gap between national and local-level action’, and noted that ‘overall progress at the community level is very limited’ (5). The HFA pointed out the challenge of establishing an action-oriented framework that strengthens local-level action, while raising community awareness and involvement. Reflecting on the lessons learned from the HFA, the Sendai Framework was established with the aim of achieving a substantial level of risk reduction and reducing the range of disaster-related losses, including the losses of human lives and livelihoods (2); it recommended the implementation of an action-oriented framework.

Although the need for the implementation of an action-oriented framework was raised, the Sendai Framework did not lay enough stress on schools, which we believe to be a core component of any disaster response. In addition, the Sendai Framework did not inherit the safe school concept, which was an important concept of the HFA. As we all know, disasters cannot be completely avoided and the associated damage cannot be easily alleviated. We would therefore like to focus on the means of achieving a reduced level of risk through school health framework. In particular, the fourth priority in the Sendai Framework, the enhancement of disaster preparedness for an effective response and to ‘build back better’ in recovery, rehabilitation, and reconstruction, deserves focus. It is our opinion that this priority can be achieved through DRR in schools, especially with the promotion of safety education, through which we can establish the norms for DRR in society.

Our principal focus is on the school health framework as defined in Focusing Resources for Effective School Health (6). Schools are appropriate for facilitating DRR because the typical school includes a policy framework, environmental arrangement, health services, and health education, which includes basic evacuation training and capacity development for students, and professional education for teachers, including hazard map development, evacuation planning, and school policy development.

## The functions of community schools in coping with disasters

To fill the gap between national- and local-level action, which was recognized in the implementation of the Hyogo Framework, we stress the importance of the functions of schools in the community. Schools perform several crucial functions, including 1) serving as a sustainable source for the dissemination of current disaster-related information and human capacity building, 2) serving as a participatory education hub among communities, and 3) being a landmark place for evacuation. We examined how these functions link DRR and the school health approach to identify further challenges.

### Schools as a sustainable source of disaster-related information and human capacity building

Needless to say, the lessons learned from previous disasters should be handed on to the next generation. Case reports from 13 different countries on DRR in school health concluded that the learning and teaching approaches in addressing DRR tend to be limited in application, and that the professional development of teachers in relation to DRR should be reconsidered, because there are only limited numbers of examples of successful interactive, inquiry-based, experiential, and action-based learning. In a number of cases, teachers were only given a manual for teaching DRR and were not provided with training. If, by any chance, training was given, then it was usually only a short-term, one-off event without any follow-up or learning reinforcement. Therefore, it is necessary to develop a training program that is significantly more systematized, reinforced, and sustainable (7).

One good example that we can report is about the 3,000 students who survived the tsunami in Japan's Great Eastern Tohoku disaster on March 13, 2013, as a result of repeated evacuation drills (8). These drills were organized based on local traditions, ‘*Tsunami Tendenko*’, in which the lesson of quick evacuation without waiting for others, including close family members, after big earthquakes (in order to avoid tsunami) was spread by word of mouth (9).

Schools can play an important role in the transmission of these lessons through the provision of repeated drills and training for disaster preparedness by schoolteachers and through the sharing of hazard maps with the surrounding schools. The initiative of experienced teachers can transform the contents of training to provide repeated drills in a manner that is culturally familiar. For experienced teachers, the development of training materials is an easier task than creating a manual with fictitious disaster scenarios. Well-localized DRR education curricula may facilitate the students’ understanding of the importance of harmony between development and the ecosystem. Teachers can adapt the training content toward the development of their students and to address local needs by themselves. School health can thereby provide a comprehensive framework for training. In this way, schools and school health have the potential to assist communities in coping with disasters.

### A participatory education hub among communities

Schools usually have links with the residents and students from surrounding communities. School health can provide a good example of participatory education (10). Thus, activities such as disaster training and the dissemination of DRR information can turn school students into catalysts and initiators, with the content of preparedness activities being transferred to parents and adults, and finally to the communities themselves (10, 11). In this way, school health and DRR methods have the capacity to transform schools into a knowledge and information hub within the community.

### A landmark place for evacuation

Displaced people require shelter, and schools can provide this, along with water, sanitation, and first aid materials (12). Though their availability may depend on the type of disaster, the idea of managing materials can be provided through school health education. School buildings may not only provide children with protection and access to education, but they may also serve as a social safety net for communities.

## Exploration of other international agendas

Referring to other recent international disaster agendas, some strategies and guidelines for strengthening DRR were published by the United Nations and their development partners. They point out the importance of establishing a national-level comprehensive school disaster management plan for child safety and protection with continuous education (12). In addition, in 2007, the Bangkok Action Agenda on school education and DRR recommended, ‘the encouragement of education departments for the development of a concrete policy for integrating disaster risk reduction into school curricula’, ‘the encouragement of education departments for the development of a concrete policy for integrating disaster risk reduction into school curricula’, and the ‘strengthening of participatory mechanisms to inform formal and non-formal DRR education’, while taking indigenous knowledge into account (13). The same document also recommends the extension of DRR education from schools to communities with the involvement of parents and to reach out to children who are out of school, including children with disabilities. We believe that these viewpoints are of importance for the purpose of DRR, because we should focus on all members of the community, including the vulnerable populations (14).

## Conclusions

Reflecting on these recommendations and our discussion, we suggest that the existing challenges and the relevant measures are as follows: 1) develop a policy of linkage between DRR and school health, 2) develop an experiential and action-based training program and materials based on current information that is drawn from the lessons of previous disasters, and 3) disseminate systematized training to the schools and the communities. In conclusion, schools should be linked with school health to provide good opportunities for DRR, with a focus on school health policy development and a strengthened participatory and community-oriented approach, involving the parents and children in communities.
